# Up-Regulation and Profibrotic Role of Osteopontin in Human Idiopathic Pulmonary Fibrosis

**DOI:** 10.1371/journal.pmed.0020251

**Published:** 2005-09-06

**Authors:** Annie Pardo, Kevin Gibson, José Cisneros, Thomas J Richards, Yinke Yang, Carina Becerril, Samueal Yousem, Iliana Herrera, Victor Ruiz, Moisés Selman, Naftali Kaminski

**Affiliations:** 1Facultad de Ciencias, Universidad Nacional Autónoma de México, Mexico City, Mexico,; **2**The Dorothy P. and Richard P. Simmons Center for Interstitial Lung Diseases, Pulmonary Allergy and Critical Care Medicine, University of Pittsburgh, Pittsburgh, Pennsylvania, United States of America,; **3**Instituto Nacional de Enfermedades Respiratorias, Mexico City, Mexico,; **4**Department of Pathology, University of Pittsburgh, Pittsburgh, Pennsylvania, United States of America; National Heart and Lung InstituteUnited Kingdom

## Abstract

**Background:**

Idiopathic pulmonary fibrosis (IPF) is a progressive and lethal disorder characterized by fibroproliferation and excessive accumulation of extracellular matrix in the lung.

**Methods and Findings:**

Using oligonucleotide arrays, we identified *osteopontin* as one of the genes that significantly distinguishes IPF from normal lungs. Osteopontin was localized to alveolar epithelial cells in IPF lungs and was also significantly elevated in bronchoalveolar lavage from IPF patients. To study the fibrosis-relevant effects of osteopontin we stimulated primary human lung fibroblasts and alveolar epithelial cells (A549) with recombinant osteopontin. Osteopontin induced a significant increase of migration and proliferation in both fibroblasts and epithelial cells. Epithelial growth was inhibited by the pentapeptide Gly-Arg-Gly-Asp-Ser (GRGDS) and antibody to CD44, while fibroproliferation was inhibited by GRGDS and antibody to α_v_β_3_ integrin. Fibroblast and epithelial cell migration were inhibited by GRGDS, anti-CD44, and anti-α_v_β_3_. In fibroblasts, osteopontin up-regulated tissue inhibitor of metalloprotease-1 and type I collagen, and down-regulated matrix metalloprotease-1 (MMP-1) expression, while in A549 cells it caused up-regulation of MMP-7. In human IPF lungs, osteopontin colocalized with MMP-7 in alveolar epithelial cells, and application of weakest link statistical models to microarray data suggested a significant interaction between osteopontin and MMP-7.

**Conclusions:**

Our results provide a potential mechanism by which osteopontin secreted from the alveolar epithelium may exert a profibrotic effect in IPF lungs and highlight osteopontin as a potential target for therapeutic intervention in this incurable disease.

## Abstract 2

Osteopontin may have a critical role in the pathogenesis of idiopathic pulmonary fibrosis, and be a target for therapeutic intervention in this disease.

## Introduction

Idiopathic pulmonary fibrosis (IPF) is a chronic fibrosing interstitial pneumonia of unknown etiology characterized by alveolar epithelial cell injury/activation, fibroblast proliferation, and exaggerated accumulation of extracellular matrix in the lung parenchyma [1,2]. The disease is usually progressive and largely unresponsive to corticosteroid and immunosuppressive therapy [2,3].

A distinctive morphological feature of IPF is the development of fibroblastic/myofibroblastic foci, represented by widely scattered, small aggregates of subepithelial mesenchymal cells immersed within a myxoid-appearing extracellular matrix [4]. These fibroblastic/myofibroblastic foci represent areas of active fibrogenesis and play a crucial role in the progressive fibrotic response [1,5]. These sites are characterized by inappropriate re-epithelialization and impaired extracellular matrix remodeling. Although significant advances have been made in the characterization of the clinical and morphological features of this disease, the molecular mechanisms that underlie IPF pathogenesis in humans are still largely unknown [1,4–7].

Osteopontin (also termed secreted phosphoprotein 1) is a phosphorylated acidic glycoprotein that contains an Arg-Gly-Asp motif that binds to the integrin family of adhesion molecules [8]. It functions as a cell adhesion and migration molecule that can bind to several ligands, including α_v_β_3_ integrin, some CD44 isoforms, and fibronectin [9,10]. Osteopontin has been implicated in a number of physiological and pathological processes including bone resorption, malignant transformation, and metastasis [11,12]. It is also considered a key cytokine regulating inflammation, cellular immune response, and tissue repair, with a unique effect on T cell function [11,12].

Using oligonucleotide microarrays we previously demonstrated that osteopontin is highly up-regulated in bleomycin-induced lung fibrosis in mice, and we reported similar results in a preliminary report involving five IPF lungs and four control samples [13,14]. Interestingly, osteopontin seems to have a profibrotic effect in the development of bleomycin-induced pulmonary fibrosis [15]. After bleomycin instillation, *osteopontin*-null mice developed reduced lung fibrosis characterized by dilated distal air spaces with decreased active transforming growth factor-beta 1 (TGF-β1) and reduced type I collagen expression compared with wild-type controls [16]. The mechanisms by which osteopontin, a cytokine with primarily TH1 effects (i.e., antifibrotic) on T lymphocytes [11,17,18], may cause profibrotic effects are not fully understood.

In this study, we applied microarrays to analyze gene expression patterns in a larger cohort of IPF lungs (13 IPF samples and 11 controls), and we analyzed the direct effects of osteopontin on human lung fibroblasts, alveolar epithelial cell migration and proliferation, and *matrix metalloprotease (MMP)* gene expression in vitro.

## Methods

### Study Population

Patients from the National Institute of Respiratory Diseases, Mexico City, México (Protocol S1), and the University of Pittsburgh, Pittsburgh, Pennsylvania, United States (Protocol S2), were included in this study. The protocol was approved by both Institutions, and written informed consent was obtained where required. Samples for oligonucleotide microarray were obtained from the tissue bank of the Department of Pathology at the University of Pittsburgh. The use of archived tissue has been approved by the local Institutional review board. Diagnosis of IPF was supported by history, physical examination, pulmonary function studies, chest HRCT, and bronchoalveolar lavage (BAL) findings, and was corroborated by open lung biopsy. The morphologic diagnosis of IPF was based on typical microscopic findings consistent with usual interstitial pneumonia [4]. The patients fulfilled the criteria of the American Thoracic Society and European Respiratory Society [7]. BAL samples were obtained at first consult as part of the initial diagnostic work-up. None of the patients had been treated with corticosteroids or immunosuppressive drugs at the time of BAL. Demographic data, pulmonary function data, and BAL differential cell counts are provide in Table 1.

**Table 1:**
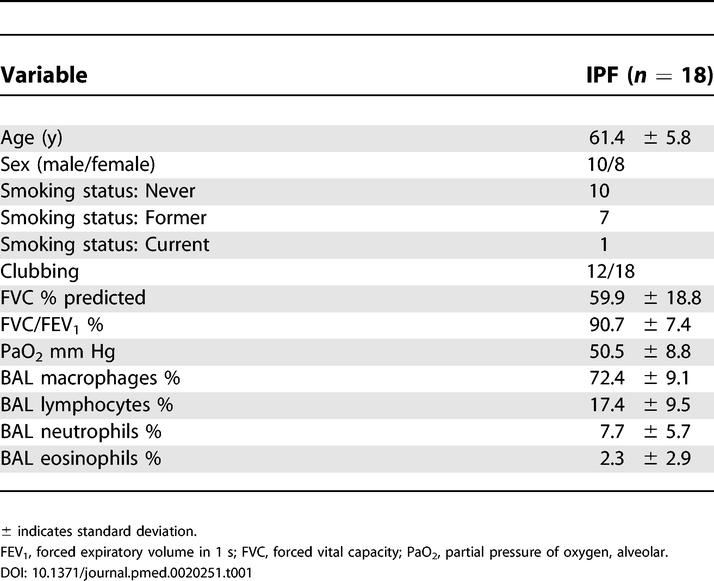
Patient Characteristics

Levels of osteopontin in BAL fluids were evaluated in ten healthy individuals (two current smokers, two former smokers, and six that had never smoked). All had normal chest X-rays and spirometries. Likewise, histologically normal lung tissues obtained at necropsy from six nonsmoking adult individuals who had died of causes unrelated to lung diseases were utilized for immunohistochemistry. For oligonucleotide microarrays, control samples included normal histology lung samples resected from patients with lung cancer obtained from the Pittsburgh Tissue Bank (Pittsburgh, Pennsylvania, United States).

### Oligonucleotide Microarrays

Surgical remnants of biopsies or lungs explanted from patients with IPF that underwent pulmonary transplant were the sources of 13 IPF samples. Lung samples resected from patients with lung cancer, obtained from the tissue bank of the Department of Pathology at the University of Pittsburgh, were the sources of 11 normal samples. None of these samples had been included in our previous study. Total RNA was extracted and used as a template to generate double-stranded cDNA and biotin-labeled cRNA, as recommended by the manufacturer of the arrays and previously described [11]. Fragmented cRNA was hybridized to Codelink Uniset I slides. After hybridization, arrays were washed and stained with streptavidin-AlexaFluor 647. The arrays were scanned using a Genepix 4000B microarray scanner. Images were analyzed using Codelink expression II analysis suite. They were visually inspected for defects and quality control parameters as recommended by the manufacturer. Data files were imported into a microarray database and linked with updated gene annotations using SOURCE (http://genome-www5.stanford.edu/cgi-bin/SMD/source/sourceSearch) and then median scaled. Based on our previous experience, all expression levels below 0.01 were brought to 0.01. Statistical analysis was performed using Scoregene gene expression package (http://www.cs.huji.ac.il/labs/compbio/scoregenes, and data visualization was performed using Genexpress (http://genexpress.stanford.edu) and Spotfire Decision Site 8.0 (Spotfire, Göteborg, Sweden). The complete set of gene array data has been deposited in the Gene Expression Omnibus database with GEO serial accession number GSE2052 (http://www.ncbi.nlm.nih.gov/geo) according to MIAME guidelines. The general approach to analysis was previously described by us [19]. Weakest link models [20,21] were fitted using the Weaklink package for the R statistical software system (http://www.r-project.org). The *p*-value was obtained from fitting a logistic regression model with a single independent variable that is the minimum of the percentiles for the expression levels for the two genes. A Bonferroni correction was applied for multiple testing.

### Bronchoalveolar Lavage

BAL was performed through flexible fiberoptic bronchoscopy under local anesthesia. Briefly, 300 ml of normal saline was instilled in 50-ml aliquots, with an average recovery of 60%–70%. The recovered BAL fluid was centrifuged at 250 *g* for 10 min at 4 °C. The cell pellet was resuspended in 1 ml of PBS and an aliquot was used to evaluate the total number of cells. Other aliquots were fixed in carbowax, stained with hematoxylin and eosin, and used for differential cell count. Supernatants were kept at −70 °C until use.

### ELISA

Quantification of osteopontin was performed in BAL fluid samples from 18 IPF patients and 10 healthy individual controls, by using a commercial sensitive and specific ELISA following the instructions of the manufacturer (Calbiochem, La Jolla, California, United States).

### Immunohistochemistry

Tissue sections were deparaffinized, rehydrated, and then blocked with 3% H_2_O_2_ in methanol for 30 min, then antigen was retrieved with citrate buffer (10 mM, pH 6.0) for 5 min in a microwave. Rabbit polyclonal antibody to human osteopontin (2 ng/ml; Calbiochem) was applied and samples were incubated at 4 °C overnight. A secondary biotinylated anti-immunoglobulin followed by horseradish peroxidase-conjugated streptavidin (BioGenex, San Ramon, California, United States) was used according to manufacturer's instructions. AEC (BioGenex) in acetate buffer containing 0.05% H_2_O_2_ was used as substrate [15,19]. The sections were counterstained with hematoxylin. The primary antibody was replaced by non-immune serum for negative control slides.

### Two-Color Immunofluorescence Analysis

A standard two-stage double-immunofluorescence labeling technique was used. Briefly, frozen sections were washed in PBS (0.01 M [pH 7.4]) for 5 min and then fixed in cold acetone for 10 min, twice. Tissues were incubated in blocking buffer (1% BSA, 5% normal serum, 0.05% NP-40, in PBS) for 30 min. The slides were then incubated with a rabbit antibody to osteopontin (1:100; Abcam, Cambridge, Massachusetts, United States) for 1 h at room temperature and washed in PBST (0.05% Tween-20 in 0.01 M PBS [pH 7.4]) for 10 min, three times. A mouse anti-MMP-7 monoclonal antibody (1:1,000; Chemicon International, Temecula, California, United States) was added and the slides were incubated for an additional 1 h at room temperature. After three 10-min washes in PBST, slides were incubated with secondary antibodies (sheep anti-rabbit IgG-Cy3, 1:1,000; and goat anti-mouse IgG-FITC, 1:1,000; Sigma-Aldrich, St. Louis, Missouri, United States) for 30 min. Slides were then washed in the same buffer and mounted with antifade medium (containing DAPI to stain cell nuclei).

### Cell Culture

Primary human normal lung fibroblasts were obtained as previously described [22], and the A549 cell line was obtained from ATCC (Rockville, Maryland, United States).

### Growth Rate Assay

Lung fibroblasts or A549 cells were seeded in 96-well culture plates at a cell density of 7.5 × 10^3^ and 5 × 10^3^ cells/well respectively, and incubated in Ham's F-12 and DMEM media, respectively (GIBCO-BRL, Grand Island, New York, United States), supplemented with 10% FBS at 37 °C in 5% CO_2_ and 95% air. After 12 h, the medium was replaced by medium with 0.1% FBS alone or 0.1% FBS plus increasing concentrations of osteopontin (0.4, 1, and 2 μg/ml) and the cells were maintained in culture for another 48 h. Cell growth was determined using the cell proliferation reagent WST-1 (Boehringer Mannheim, Mannheim, Germany) as previously described [19]. All assays were performed in triplicate. In parallel experiments, fibroblasts and A549 cells were pretreated for 25 min with antibody to human α_v_β_3_ integrin (10 μg/ml; Chemicon), the pentapeptide Gly-Arg-Gly-Asp-Ser (GRGDS; Calbiochem), or antibody to human CD44 (NeoMarkers, Fremont, California, United States), and then osteopontin was added (2 μg/ml). Additionally, A549 cells were pretreated with antibody to human epidermal growth factor receptor (EGFR; 10 μg/ml; Chemicon).

### Cell Migration Assay

Migration of fibroblasts and A549 cells was assayed using commercially available 24-well collagen-coated Boyden chambers (Chemicon) with an 8-μm pore size. Briefly, a semi confluent (∼80%) monolayer of lung fibroblasts or A549 cells was harvested with trypsin-EDTA, centrifuged, and resuspended in Ham's F-12 medium containing 5% BSA. The cell suspensions (3 × 10^5^ cells/well) were added to the upper chamber. The lower chamber contained 0.3 ml of medium with 5% BSA alone or with 10 μg/ml of recombinant human osteopontin (Calbiochem). PDGF (8 ng/ml) and epidermal growth factor (EGF; 50 ng/ml) were used as positive controls for fibroblasts and for A549 cells, respectively. Additional BSA-coated chambers were used as blanks for each sample. After incubation for 8 h at 37 °C in a humidified incubator with 5% CO_2_ and 95% air, the nonmigrating cells on the top of Boyden chamber were scraped and washed. The migrating cells were quantitated according to manufacturer's instructions. Briefly, the cells were stained and the color eluted with 300 μl of extraction buffer, and 150-μl aliquots were measured in an ELISA plate reader at 545 nm. All assays were performed in duplicate. In parallel experiments, A549 cells and fibroblasts were pretreated as described above for growth rate.

### RNA Isolation and Northern Blot Analysis

Total RNA was extracted from lung fibroblasts and A549 epithelial cells using the RNeasy Mini Kit (Qiagen GmbH, Germany). Cells were lysed and homogenized in the presence of a highly denaturing guanidine isothiocyanate-containing buffer. The samples were then applied to an RNeasy minicolumn, and RNA was eluted in 30 μl of water. Total RNA (20 μg/lane) was fractionated on a 1% agarose gel containing 0.66 M formaldehyde [21]. Ribosomal RNA was visualized with ethidium bromide, and the fractionated RNA was transferred onto a Nytran transfer membrane (NEN Life Science Products, Boston, Massachusetts, United States) by capillary blotting overnight. RNA was immobilized by baking at 80 °C for 2 h, and then prehybridized at 42 °C for 18 h in 5× SSC, 50% formamide, 5× Denhardt's solution, and 0.5% SDS, containing 100 μg/ml of denatured salmon sperm DNA. Hybridization was carried out at 42 °C for 18 h in hybridization buffer composed of formamide, 0.5% SDS, and heat-denatured ^32^P-labeled cDNA probes. After washing, membranes were dried and exposed to Kodak BIOMAX MS film at −70 °C with an intensifying screen. The cDNA clones for human MMP-1, TIMP-1, α1 type I collagen, and RNA ribosomal 18S were obtained from ATCC. Human MMP-7 cDNA was a kind gift of Lynn Matrisian, Vanderbilt University, Nashville, Tennessee, United States. The cDNA probes were radiolabeled with ^32^P-dCTP to a specific activity of 200 × 106 dpm/μg using a Random Primer Labeling Kit (Stratagene, La Jolla, California, United States). All experiments were repeated twice.

### Quantitative Real-Time RT-PCR

1 μg of RNA was treated with 1 unit of DNAase (Life Technologies, Grand Island, New York, United States). First-strand cDNA was synthesized by reverse transcription with random primers and Moloney-murine leukemia virus reverse transcriptase according to manufacturer's protocol (Advantage RT-for-PCR Kit; Clontech, Palo Alto, California, United States).

Real-time PCR amplification was performed using i-Cycler iQ Detection System (BioRad, Hercules, California, United States), using TAQMAN probes (PE Applied Biosystems, Wellesley, California, United States) labeled with FAM and TET. PCR was performed with the cDNA working mixture in a 25-μl reaction volume containing 3 μl of cDNA, 20 mM Tris-HCl (pH 8.3), 50 mM KCl, 2 mM MgCl_2_, 200 μM dNTPs, 0.2 μM specific 5′ and 3′ primers for 18S rRNA, 0.6 μM specific 5′ and 3′ primers for gene target, 0.2 μM of each probe TAQMAN (18S rRNA and gene target), and 1.25 units of AmpliTaq GOLD DNA polymerase (PE Applied Biosystems). A dynamical range was built with each product of PCR on copy number serial dilutions from 1 × 10^8^ to 1 × 10^1^; all PCRs were performed in triplicate. Standard curves were calculated referring the threshold cycle (Ct) to the log of each cDNA dilution step. Results were expressed as the number of copies of the target gene normalized to 18S rRNA. Some primers used in PCR reactions were designed using Beacon Designer software 2.1 (BioRad) and checked for homology in BLAST. The cycling conditions for PCR amplification were performed using the following protocol: Initial activation of AmpliTaq Gold DNA polymerase at 95 °C for 7 min; and 40 cycles of denaturation at 95 °C for 30s, annealing at 60 °C for 30 s, and extension at 72 °C for 30 s. The sequences of the PCR primer pairs and probes for each gene are shown in Table 2. All PCR experiments were done in duplicate.

**Table 2:**
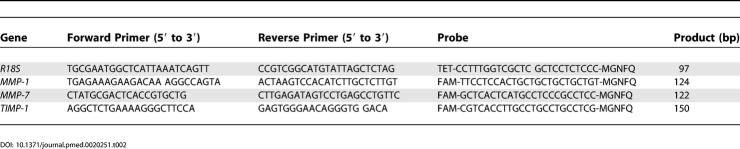
Primers and Probes for Real-Time PCR

### PAGE Zymography

For MMP-7 analysis, conditioned media was electrophoresed in 12.5% SDS gels containing as substrate bovine CM-transferrin (0.3 mg/ml) and heparin [22]. After electrophoresis, gels were washed in a solution of 2.5% Triton X-100, and incubated overnight at 37 °C in 100 mM glycine (pH 7.6), plus 10 mM CaCl_2_ and 50 nM ZnCl_2_. Identical gels were incubated but in the presence of 20 mM EDTA. Gels were stained with Coomassie Brilliant Blue R250 and destained in a solution of 7.5% acetic acid and 5% methanol. Recombinant human MMP-7 (Chemicon) was used as positive control.

### Western Blot Analysis

Serum-free conditioned media was centrifuged at 300 *g* at 4 °C for 30 min to remove cell debris, then concentrated by lyophilization. Samples were solubilized in water, and aliquots containing 5 μg of protein were mixed with Laemmli sample buffer and electrophoresed on 10% or 12.5% SDS-polyacrylamide gels. Proteins were transferred to nitrocellulose filters at 15 V for 20 min using semi-dry transfer cell (BioRad). Nonspecific sites were blocked overnight with 4% (w/v) nonfat dried milk in PBS, and membranes were incubated with rabbit antibody to human interstitial collagenase IgG (1:250 in PBS with 1% BSA; Santa Cruz Biotechnology, California, United States), antibody to human TIMP-1 (Chemicon), antibody to human MMP-7 (Oncogene, San Diego, California, United States), or monoclonal antibody to human α-smooth muscle actin (2 μg/ml; Cymbus Biotechnology, Chandlers Ford, Hants, United Kingdom) for 2 h at room temperature. Filters were incubated with secondary antibody conjugated to peroxidase for 1 h at room temperature. Finally, the filters were developed with the Enhanced Chemiluminescence detection system (Amersham Pharmacia Biotech, Piscataway, New Jersey, United States) using radiograph film (Hyperfilm, Amersham Pharmacia Biotech) according to the instructions of the manufacturer. Western blots were repeated twice.

## Results

### *Osteopontin* Gene Expression Is Highly Up-Regulated in IPF Lungs

**Figure 1:**
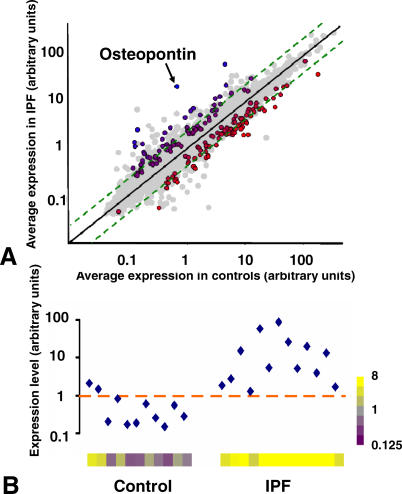
Osteopontin Expression Levels by Microarray Analysis from Controls and IPF Lungs Total RNA was used to generate double-stranded cDNA and biotin-labeled cRNA. Fragmented cRNA was hybridized to Codelink Uniset I slides and stained and scanned as described in Materials and Methods. (A) A log scale scatter plot of the average of intensity of all the genes on the arrays in controls (x-axis) and IPF (y-axis). Colored points indicate 178 genes that were significantly changed (*p* < 0.01 in TNoM and Student's t-test). Points are colored by their fold ratios; progressive shades of blue indicate increase, and progressive shades of red indicate decrease. Points colored in gray did not reach significance. Oblique solid line indicates the line of equality. Two green dashed lines are lines that depict 10-fold change. (B) Osteopontin levels in individual samples are shown. The y-axis is expression level in arbitrary fluorescence levels (log scale). The blue quadrangles are osteopontin levels in individual samples. Heat map shows sample osteopontin levels normalized to the geometric mean of osteopontin in controls and log base 2-transformed.

The complete microarray dataset is available at the Gene Expression Omnibus database with GEO serial accession number GSE2052 (http://www.ncbi.nlm.nih.gov/geo). Gene expression patterns clearly distinguished IPF lungs from normal lungs. *Osteopontin* was the most up-regulated gene among the genes that distinguished IPF lungs (Figure 1A). Osteopontin levels were increased more than 20-fold (mean expression 0.62 in control lungs, 16.8 in IPF lungs; Figure 1B). This increase was statistically significant whether we applied threshold number of misclassifications (TNoM) score (*p* = 0.000221), or Student's t-test (*p* = 0.0000035). We corrected for multiple testing by controlling the false discovery rate at the 5% level [23]. Osteopontin expression levels were higher in most IPF lungs than in any control lung (Figure 1B).

### Osteopontin Is Increased in BAL Fluid from IPF Patients

**Figure 2:**
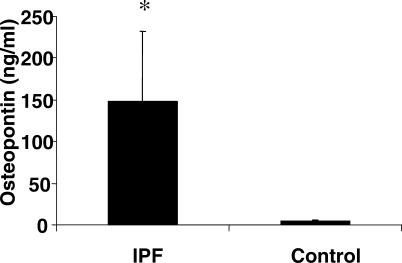
Osteopontin Levels in BAL Fluid Quantification of osteopontin by ELISA was performed in BAL fluid samples from 18 IPF patients and 10 healthy individual controls. An increased concentration of soluble osteopontin was found in the BAL fluid obtained from IPF patients compared with healthy controls. The data represent the mean ± standard deviation (SD). * *p* < 0.01.

Osteopontin protein was quantified in BAL fluids from 18 IPF patients and 10 healthy controls. As shown in Figure 2, ELISA measurement revealed a significant increase in the immunoreactive protein in the fluids derived from IPF lungs (148.8 ± 83 ng/ml in IPF lungs versus 3.8 ± 2.0 ng/ml in healthy controls, *p* < 0.01).

### Immunoreactive Osteopontin Is Localized Primarily in Epithelial Cells

**Figure 3:**
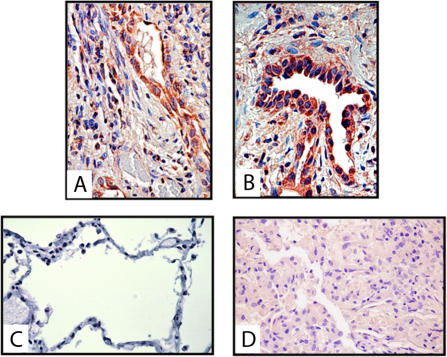
Localization of Osteopontin in IPF Lungs Immunoreactive protein was revealed with AEC, and samples were counterstained with hematoxylin. Two representative IPF lung samples exhibited strong epithelial staining (original magnification, 40×) (A,B). Control lung showed no staining (C). Negative control section from IPF lung in which the primary antibody was replaced with nonimmune serum also showed no staining (40×) (D).

To examine the cellular source of osteopontin we analyzed IPF and control lungs by immunohistochemistry. As illustrated in Figure 3A–3C, osteopontin was localized primarily in alveolar epithelial cells that exhibited an intense cytoplasmic staining in IPF lungs. Occasionally, clusters of alveolar macrophages were also positive (Figure 3C). Immunohistochemical staining for osteopontin was negative in normal lungs as well as in lung tissue samples incubated with nonimmune sera (Figure 3D).

### Osteopontin Induces Fibroblast and Epithelial Cell Growth Rate

To determine the effect of osteopontin on the growth rates of fibroblasts and epithelial cells, cells were stimulated with increasing concentrations of osteopontin, and the cell number was determined after 48 h using the cell proliferation reagent WST-1. Significant dose-dependent increases in cell proliferation were observed with 1 μg/ml and 2 μg/ml. Two different fibroblast lines reached 220% and 380% over controls (*p* < 0.01), while in two different experiments A549 cell lines exhibited 60% and 80% increases of growth rate over control (*p* < 0.01).

**Figure 4:**
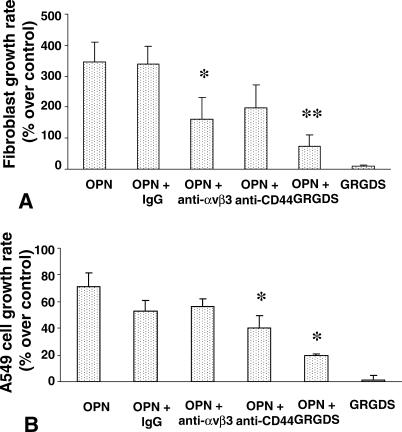
Effect of Osteopontin on Fibroblasts and Epithelial Cell Proliferation Human normal lung fibroblasts (A) and A549 epithelial cells (B) were grown in Ham's F-12 medium with 0.1% FBS and stimulated with 2 μg/ml osteopontin. In parallel, osteopontin-stimulated cells were treated with anti-α_v_β_3_, anti-CD44, and GRGDS. Each bar represents the mean ± SD of three experiments performed in triplicate; *p < 0.05; **p < 0.01. OPN, osteopontin

Osteopontin-induced fibroblast proliferation (2 μg/ml) was significantly suppressed by GRGDS-pentapeptide, which interrupts binding of RGD-containing proteins to cell surface integrins (*p* < 0.01) and by antibody to α_v_β_3_ integrin (*p* < 0.05), suggesting that the effect of osteopontin on growth rate was mediated by the interaction of the GRGDS domain of osteopontin with α_v_β_3_ integrin (Figure 4A). In contrast, epithelial cell growth was partially inhibited by antibody to CD44 (*p* < 0.05) (Figure 4B). Epithelial cell growth was also significantly suppressed by GRGDS but not by antibodies to α_v_β_3_ (Figure 4B).

### Osteopontin Induces Fibroblast and Epithelial Cell Migration

**Figure 5:**
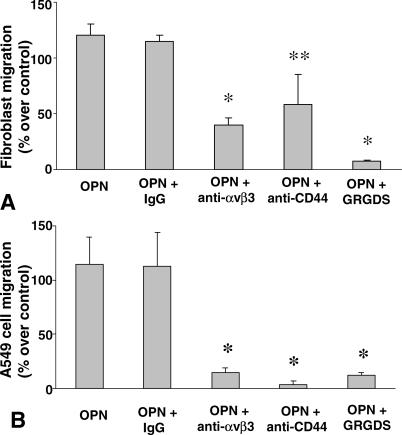
Effect of Osteopontin on Fibroblasts and Epithelial Cell Migration Fibroblasts (A) and A549 epithelial cells (B) were placed in the upper compartment of a Boyden-type chamber, and Ham's F-12 medium containing 5% BSA alone or with 10 μg/ml of osteopontin was added to the lower compartment. After 8 h of incubation, the migrating cells were stained, and the absorbance of the stained solution was measured by ELISA. In parallel experiments, osteopontin-stimulated cells were treated with anti-α_v_β_3_, anti-CD44, and GRGDS. Each bar represents the mean ± SD of three experiments; *p < 0.01; **p < 0.05. OPN, osteopontin

To examine the effect of osteopontin on cell migration, we used collagen-coated Boyden chambers, a well-established in vitro assay system. The number of cells that migrated in absence of osteopontin was used as control (0% migration). As revealed in Figure 5A, fibroblasts significantly moved toward osteopontin compared with cells exposed to medium plus 5% BSA alone in the lower chamber. Osteopontin (10 μg) enhanced fibroblast migration by 120% ± 11% (*p* < 0.01), an enhancement similar to that obtained with the potent fibroblast mitogen platelet-derived growth factor, used as a positive control (157% ± 12%, unpublished data). To analyze possible mechanisms by which osteopontin stimulates migration, different blockers were used. Fibroblast migration was significantly reduced by GRGDS and antibody to α_v_β_3_ integrin (*p* < 0.01), and by antibody to CD44 (*p* < 0.05). The inhibition of cell migration was specific, since incubation with IgG had no effect (unpublished data).

A549 lung cells also showed a significant increase in cell migration in response to osteopontin (Figure 5B). After 8 h of incubation, A549 cells passing through the membrane increased by 114% ± 25% compared to control cells (*p* < 0.01). Epidermal growth factor, used as a positive control, induced a 168% ± 14% increase in cell migration (unpublished data). Osteopontin-induced migration was abolished when the epithelial cells were pretreated individually with GRGDS, anti-α_v_β_3_ integrin, or anti-CD44 (*p* < 0.01; Figure 5B).

### Osteopontin Induces an Environment That Favors Extracellular Matrix Deposition in Fibroblasts

#### MMP-1 expression by fibroblasts

**Figure 6:**
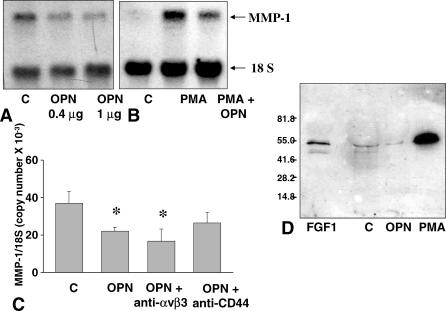
Effects of Osteopontin on MMP-1 Gene and Protein Expression in Two Human Lung Fibroblast Cell Lines (A) Representative Northern blot of 20 μg total cellular RNA per lane extracted from control cells and fibroblasts stimulated with 0.4 μg/ml and 1 μg/ml osteopontin. Both concentrations of osteopontin induced a down-regulation in the expression of MMP-1. (B) Osteopontin also reduced overexpression of MMP-1 in APMA-stimulated cells. (C) The expression level of MMP-1 by real-time PCR was determined as described in Materials and Methods and normalized to the level of 18S ribosomal RNA. In parallel experiments, osteopontin-stimulated cells were treated with anti-α_v_β_3_ and anti-CD44. Bars represent mean ± SD (*p < 0.05). (D) Representative Western blot demonstrating a decrease of immunoreactive MMP-1 in conditioned media from fibroblasts stimulated with osteopontin. Fibroblasts treated with APMA and FGF-1 plus heparin used as positive controls strongly induced MMP-1 expression. C, control; FGF1, FGF-1 plus heparin; OPN, osteopontin; PMA, APMA-stimulated.

Under basal conditions, some primary human lung fibroblast cell lines may express MMP-1, while others express the enzyme only when stimulated, for example, by aminophenylmercuric acetate (APMA). In this context, the effect of osteopontin on MMP-1 expression was examined by Northern blot analysis in a cell line producing MMP-1 under basal conditions, and in a cell line that did not produce MMP-1 but was stimulated by APMA (Figure 6A and 6B). Osteopontin down-regulated both the basal and the APMA-induced MMP-1 transcript level. When the signal of MMP-1 mRNA was normalized to the level of 18S RNA and quantified by densitometry, a reduction of ∼50% was noticed. This result was confirmed by real-time PCR that showed ∼40% inhibition (*p* < 0.05; Figure 6C). Inhibition of MMP-1 expression was partially blocked by antibody to CD44, while it was not affected by antibody to α_v_β_3_ integrin. The inhibitory effect of osteopontin on MMP-1 was also observed at the protein level by Western blot analysis. As shown in Figure 6D, the level of immunoreactive MMP-1 present in the conditioned medium was decreased in the fibroblasts stimulated with osteopontin as compared with control cells.

#### Osteopontin increases TIMP-1 expression by fibroblasts**

**Figure 7:**
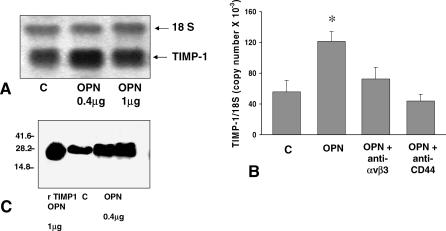
Effect of Osteopontin on TIMP-1 Gene and Protein Expression by Fibroblasts (A) Northern blot of 20 μg total cellular RNA per lane extracted from control and fibroblasts stimulated with 0.4 μg/ml and 1 μg/ml osteopontin. Both concentrations of osteopontin induced an increase of TIMP-1 expression. (B) The expression level of TIMP-1 by real-time PCR normalized to the level of 18S ribosomal RNA corroborates TIMP-1 up-regulation by osteopontin (*p < 0.01). In parallel experiments, osteopontin-stimulated cells were treated with anti-α_v_β_3_ and anti-CD44. (C) Western blot demonstrating an increase of immunoreactive TIMP-1 in conditioned media from fibroblasts stimulated with osteopontin. C, control; OPN, osteopontin.

The effect of osteopontin on *TIMP-1* gene expression in fibroblasts is illustrated in Figure 7. Northern blot analysis (Figure 7A) revealed an increase in *TIMP-1* gene expression at 0.4 μg/ml and 1 μg/ml osteopontin when compared to control. This result was confirmed by real-time PCR (Figure 7B). Osteopontin increased the TIMP-1/18S ribosomal RNA from 55.7 ± 14.2 copies to 121.2 ± 13.1 copies (*p* < 0.01). Both anti-CD44 and anti-α_v_β_3_ integrin abolished this increase. Osteopontin also increased TIMP-1 protein expression in conditioned medium as illustrated in Figure 7C.

#### Osteopontin induces collagen gene expression in fibroblasts

**Figure 8:**
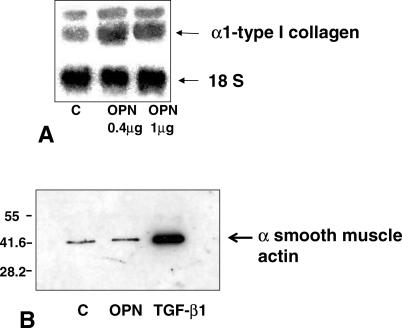
Effects of Osteopontin on Collagen Gene Expression and Smooth Muscle Alpha Actin Protein in Human Lung Fibroblasts (A) Northern blot of 20 μg total cellular RNA per lane extracted from control and fibroblasts stimulated with 0.4 μg/ml and 1 μg/ml osteopontin. Both concentrations of osteopontin induced an up-regulation in the expression of α1 type I collagen. (B) Western blot showing no effect of osteopontin on immunoreactive α smooth muscle actin. Recombinant TGF-β1 was used as a positive control. C, control; OPN, osteopontin.

The effect of osteopontin on collagen gene expression is depicted in Figure 8. Northern blot analysis (Figure 8A) revealed a 2-fold increase in *α1 type I collagen* gene expression. Expression of α-smooth muscle actin in fibroblasts was induced by TGF-β1 but not by osteopontin (Figure 8B).

### Osteopontin Increases MMP-7 Expression by Epithelial Cells

**Figure 9:**
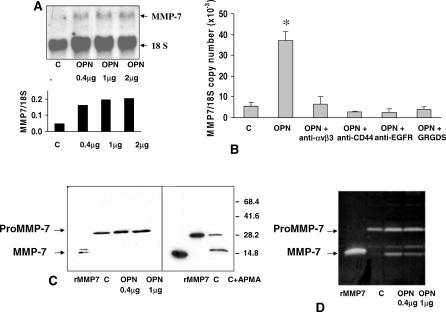
Effect of Osteopontin on MMP-7 Gene and Protein Expression and Activity in A549 Epithelial Cells (A) Northern blot of 20 μg total cellular RNA per lane extracted from control and A549 cells stimulated with 0.4, 1, and 2 μg/ml osteopontin. (B) Densitometry of Northern blot and normalization of MMP-7 to 18S demonstrates a 3- to 4-fold increase in MMP-7 over control. (C) Real-time PCR showing up-regulation of MMP-7 expression by osteopontin (*p < 0.01) and inhibition by anti-α_v_β_3_, anti-CD44, anti-EGFR, and GRGDS. (D) Western blot demonstrating an increase of immunoreactive MMP-7 in conditioned medium from A549 epithelial cells stimulated with osteopontin. Activation of pro-MMP-7 by APMA is shown in leftmost lane. (E) Zymography of conditioned media in 12.5% SDS gels containing bovine CM-transferrin (0.3 mg/ml) and heparin as substrate. C, control; OPN, osteopontin.

Stimulation of A549 cells with osteopontin (0.4 μg/ml and 2.0 μg/ml) induced an up-regulation of MMP-7 gene expression as illustrated by Northern blot in Figure 9A. Real-time PCR revealed a 6-fold increase at 6 h after osteopontin stimulation, which was abolished by treatment with anti-α_v_β_3_, anti-CD44, anti-EGFR, or GRGDS as shown in Figure 9B. Pro-MMP-7 overexpression was confirmed at the protein level by Western blot analysis of the epithelial cells conditioned medium (Figure 9C). As recombinant MMP-7 used as control showed a lower molecular weight band to that of conditioned medium, this one was treated with APMA to activate pro-MMP-7. Activated proenzyme showed a similar molecular weight band to the recombinant protein. Zymography using CM-transferrin as substrate showed that treatment of A549 epithelial cells with 0.4 μg/ml and 1 μg/ml osteopontin induced an increase of both the pro-MMP-7 and MMP-7 activity bands (Figure 9D).

### Osteopontin Colocalizes with MMP-7 in Alveolar Epithelial Cells from IPF Lungs

**Figure 10:**
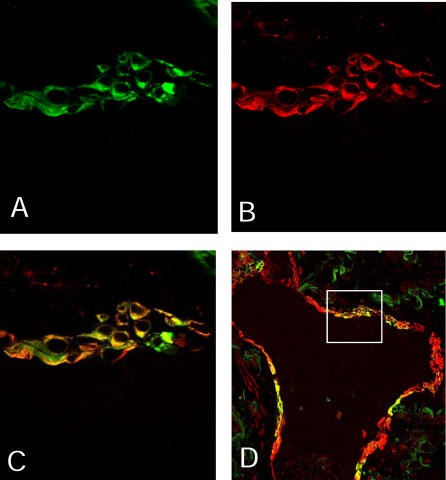
Osteopontin and MMP-7 Colocalization in IPF Lungs (A−C) MMP-7 staining is shown in green (A), osteopontin is shown as red staining (B), and overlap of staining is shown in yellow (C), suggest colocalization of MMP-7 and osteopontin in alveolar epithelial cells in IPF lungs (60×). (D) A lower-magnification image (20×) of the same region (A–C) with the same color coding. The white rectangle depicts area shown in (A−C).

Since we have previously demonstrated that IPF lungs strongly express MMP-7 in alveolar epithelial cells [14], we evaluated whether the staining pattern of this enzyme is associated with that of osteopontin. Figure 10 illustrates confocal microscopy images showing that MMP-7 (Figure 10A) is partially overlapped with that of osteopontin (Figure 10B), giving substantial double labeling (Figure 10C and 10D). Normal lungs did not exhibit any significant staining for MMP-7 or osteopontin (unpublished data).

### Weakest Link Models Identify a Statistically Significant Interaction between Osteopontin and MMP-7

**Figure 11:**
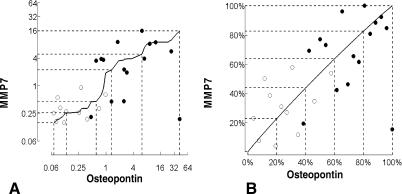
Weakest Link Models of Osteopontin and MMP-7 IPF samples are depicted in solid dots and controls in open dots; the y-axis is MMP-7 expression and x-axis is osteopontin expression. Black solid line is the curve of optimal use showing that the expression levels for MMP-7 and osteopontin jointly interact to determine the IPF phenotype. The probability contour plot is shown in terms of the observed expression data (scale is log base 2 for gene expression data) (A); and probability contour plot is shown in terms of percentiles of the data (scale is percentiles) (B).

To determine whether MMP-7 and osteopontin expression levels jointly distinguish IPF and control samples, we applied weakest link statistical models [20]. Weakest link models assume that two genes jointly impact the probability that a randomly selected sample belongs to a certain phenotype (IPF in this case) only if expression levels of the two genes lie on a low-dimensional curve whose form is specified by the model and estimated using the observed quantiles of the data. Our analysis detected a statistically significant joint effect of MMP-7 and osteopontin on the IPF phenotype (*p* < 0.001). This joint relationship is illustrated in Figure 11 for both the gene expression values (Figure 11A) and the sample percentiles (Figure 11B). To assess the relevance of this association, we chose both substantively meaningful genes and a more general set of genes to evaluate the contribution of the weakest link model relating osteopontin and MMP-7 (Figure 11). Among 14 matrix metalloproteases and their inhibitors represented on the Human Uniset I chip, osteopontin and MMP-7 exhibited the most significant biological interaction. Osteopontin also had significant interactions (weakest link models with *p* < 0.05) with MMP-1, MMP-2, and MMP-11 but none was as statistically significant as MMP-7. We also ran weakest link models combining *osteopontin* with each of 400 genes that passed the false discovery rate of less than 0.05 for differential expression between IPF and control samples. The interaction of *osteopontin* with MMP-7 in a weakest link model was more significant than 371 of these 400 genes.

## Discussion

In the present study we focused on the profibrotic effects of osteopontin, a multifunctional cytokine that mediates diverse biological functions, including cell adhesion, chemotaxis, and signaling, as well as tissue reparative processes [8,11,12]. We demonstrated that osteopontin was the most up-regulated gene in lungs of IPF patients, and that it was mainly expressed by alveolar epithelial cells. To better understand the potential local profibrotic effects of osteopontin, we studied its effects on lung fibroblasts and epithelial cells. Functionally, osteopontin induced fibroblast and epithelial cell proliferation and migration. The effect on fibroblast migration and proliferation was dependent mainly on integrins, while in epithelial cells proliferation was mainly dependent on CD44 and migration was dependent on CD44 and integrin signaling. Osteopontin exhibited profibrotic effects on molecules involved in extracellular matrix remodeling. Thus, in fibroblasts, osteopontin increased TIMP-1 and type I collagen and inhibited MMP-1 expression, while in alveolar epithelial cells it induced MMP-7. The effects on TIMP-1 and MMP-1 expression appeared to be mostly dependent on CD44, while the effect on MMP-7 expression was dependent on CD44 and integrin signaling. Interestingly, osteopontin was colocalized with MMP-7 in alveolar epithelial cells of IPF lungs, and application of the weakest link models to microarray data suggested that the genes of both interacted to affect the IPF phenotype. Our results provide a potential mechanism by which osteopontin secreted from epithelial cells exerts its profibrotic effects through direct signaling on fibroblasts and epithelial cells.

Several studies in experimental tissue fibrosis have suggested a possible profibrotic role of osteopontin. In kidney fibrosis, osteopontin enhances macrophage recruitment and stimulates the development of renal scarring after an acute ischemic insult [24]. Also, osteopontin expression is increased in the myocardium after myocardial infarction, and the lack of this mediator is associated with decreased collagen accumulation [25]. Similarly, osteopontin appears to be an important mediator of the cardiac profibrotic effects of angiotensin II by promoting collagen synthesis and remodeling in the interstitial myocardium [26]. In experimental lung fibrosis it has been suggested that osteopontin produced by alveolar macrophages functions as a fibrogenic cytokine [15,16]. In granulomatous lung diseases, osteopontin is also up-regulated and its main sources are macrophages and T lymphocytes [27,28]. We observed that in human IPF lungs, hyperplastic alveolar epithelial cells seemed to be a source of osteopontin, which is consistent with the findings of Berman et al. [15,16]. These observations support the key role of alveolar epithelial cells as regulators of the lung profibrotic microenvironment in IPF, thus emphasizing the critical difference between the mechanisms of fibrosis in IPF compared with animal models or with lung fibrosis associated with inflammatory disorders.

Positive feedback mechanisms have been previously proposed for osteopontin and MMP-2 [29], where osteopontin induces MMP-2 expression [30] and is cleaved and activated by MMP-2 [29]. The same mechanism has been proposed for osteopontin and MMP-3, where osteopontin binds and activates MMP-3, which in turn can cleave and activate it [31]. Similarly, osteopontin is cleaved and activated by MMP-7 [31], and we observe that MMP-7 is induced by osteopontin in epithelial cells, suggesting that this positive feedback mechanism is also applicable to osteopontin and MMP-7. This is also supported by the colocalization of MMP-7 and osteopontin in IPF epithelial cells, and by the computational relationship of the expression levels of osteopontin and MMP-7. Interestingly, *MMP-7* and *osteopontin* are β-catenin target genes [32,33]. Recently, Chilosi et al. reported impressive activation of WNT/β-catenin in IPF lungs [34]. They have mainly observed β-catenin nuclear localization in proliferative bronchiolar lesions, where it colocalized with MMP-7 [34]. Collectively, these results could indicate a mechanism by which osteopontin and MMP-7 are induced by aberrant activation of the WNT/β-catenin pathway, and each affects the function and expression of the other gene, thus representing a local positive feedback mechanism that facilitates a chronic, relentless lung disease.

Although intriguing, this proposed positive, self-perpetuating loop in itself cannot explain increased collagen deposition, the critical hallmark of fibrosis. In this context, our results suggest that osteopontin affects the critical balance between MMPs and their inhibitors through its cell-specific effects. In agreement with the inhibition of interleukin 1β-stimulated increases in MMP-2 and MMP-9 observed by Xie et al. [35], we observed that in human lung fibroblasts, osteopontin caused a significant reduction of baseline as well as APMA-stimulated MMP-1, an MMP responsible for fibrillar collagen degradation. Additionally, we observed a concomitant increase in TIMP-1, the main inhibitor of MMP-1 (as well as MMP-2 and −9), and in *type I collagen* gene expression, suggesting that osteopontin may facilitate a profibrotic environment not only by its effect on epithelial cells but also by inducing a nondegradative microenvironment similar to the one observed in IPF and experimental lung fibrosis [36,37]. Interestingly, osteopontin did not induce α-smooth muscle actin in lung fibroblasts in vitro, suggesting that although it had a role in facilitating the profibrotic environment in IPF, it had weaker role in the formation of myofibroblast foci. However, further experiments will be needed to determine this point.

Migration and proliferation of fibroblasts are essential for the expansion of their populations and the formation of the fibroblastic foci that seem to represent the “leading edge” of the progressive fibrotic process [5]. The observation that osteopontin induced both migration and proliferation of primary human lung fibroblasts suggests that osteopontin may be involved in this process and is supported by previous observations in murine and human fibroblast cell lines [15]. Osteopontin promotes fibroblast collagen gel contraction and rat cardiac fibroblast proliferation, and it has been suggested that it is an important mediator of angiotensin II regulation of fibroblast behavior in the cardiac remodeling process [38]. It also induced proliferation of vessel smooth muscle cells, suggesting that it is involved in vascular remodeling during the development of atherosclerosis [39,40]. These results highlight the sufficiency of a cytokine secreted from alveolar epithelial cell to induce many of the phenotypic changes associated with lung fibrosis.

The mechanisms by which osteopontin influences epithelial and fibroblast cells are not fully understood. In general it has been proposed that osteopontin affects cells by binding to CD44 isoforms, certain integrins, and EGFR [41,42]. It is unknown whether the expression of these receptors is changed in IPF; CD44 is expressed on lung fibroblasts and epithelial cells [43], is induced in radiation- and bleomycin-induced pulmonary fibrosis [44] and in acute alveolar fibrosis [45], and is critical for resolution of noninfectious lung inflammation [46]. The integrin receptors for osteopontin are widely expressed in lung epithelial cells and fibroblasts [47], but a change in their repertoire has not been reported yet with IPF. In this paper we did not seek to fully dissect these mechanisms; however, we explored some of the possible receptors that may be important to osteopontin effect on fibroblasts and epithelial cells. Our results support some of the previous observations regarding the importance of integrin-mediated signaling in fibroblast migration. Interestingly, we observed a differential effect in epithelial and fibroblasts cells. Inhibition of CD44 significantly reduced the effects of osteopontin on cell proliferation in epithelial cells, while α_v_β_3_ inhibition seemed to affect mostly fibroblasts. This integrin is a receptor for a wide variety of extracellular matrix ligands with an exposed RGD sequence, including vitronectin, fibronectin, fibrinogen, thrombospondin, proteolyzed collagen, von Willebrand factor, as well as osteopontin, and appears to play a critical role in cell migration [48]. The hyaluronic acid receptor CD44 is also a receptor for osteopontin, and it has been implicated in chemotaxis mediated by this mediator [10,49,50]. In our experiments, the pan-integrin antagonist GRGDS-pentapeptide added to the culture medium abolished the effects in fibroblasts and epithelial cells, suggesting that different integrins may be involved in both cell types, and that for some effects, both integrins and CD44 are required. Detailed studies of the effects of variant osteopontin isoforms as well as cells that express different receptors will be needed to elucidate these mechanisms.

Our study focused on human tissues and human cell lines because of the unique features of IPF that are not readily mimicked by any animal models. We insisted on using primary human lung fibroblasts in our experiments; therefore, we are confident that these results represent a mechanism that may actually occur in the human lung. Unfortunately, it is nearly impossible to work with primary alveolar epithelial cells, and we had to resort to the epithelial cell line A549. However, we present evidence obtained from human lungs that suggest that the mechanisms that we proposed in vitro do exist in the human IPF lung.

In summary, in this study we highlight the role of osteopontin in human IPF. Although previous studies have suggested that osteopontin has a potential profibrotic effect in animal models of lung fibrosis, its role in human IPF was unclear. We demonstrated that osteopontin is highly expressed in IPF lungs, and that it is primarily expressed by hyperplastic alveolar epithelial cells. We demonstrated that osteopontin affected fibroblast and epithelial cell proliferation and migration, and that it had fibrosis-relevant effects on MMP and TIMP expressions. Our results suggest a mechanism explaining most of the profibrotic effects of osteopontin by its direct effects on fibroblasts and epithelial cells in the lungs. Furthermore, our results suggest that in IPF the interaction between MMP-7 and osteopontin may be involved in the relentlessly progressive nature of the disease, and highlight osteopontin as a potential target for therapeutic intervention in this incurable disease.

## Supporting Information

Protocol S1 Sample Mexico (746 KB ZIP).

Protocol S2 Microarrays (2.1 MB ZIP).

### Accession Numbers

The GenBank (http://www.ncbi.nlm.nih.gov/) accession numbers of the genes discussed in this paper are *R18S* (X03205), *MMP-1* (NM_002421), *MMP-7* (XM_017384), and *TIMP-1* (X03124).

**Patient summary**

**Background**

Idiopathic pulmonary fibrosis is a chronic progressive disease of the lung that leads to increasing amounts of scar tissue with subsequent destruction of the lung. There is no specific cure at present. Patients may be treated with corticosteroids or drugs to suppress their immune system, although these drugs are usually not effective. Some patients receive lung transplants.

**Why Was This Study Done?**

Previous work has suggested that a protein called osteopontin is increased in mice that have lung fibrosis and that mice that do not have the gene for osteopontin are protected from lung fibrosis. The researchers wanted to investigate if osteopontin was also involved in the human disease.

**What Did the Researchers Do and Find?**

They looked at samples taken from the lungs of people with idiopathic pulmonary fibrosis and other diseases and measured many genes that are expressed there. They found that osteopontin was increased in the lungs of people with idiopathic pulmonary fibrosis. They then looked at cultures of lung cells and found that osteopontin caused an increase in the number and movement of cells that are involved in lung fibrosis. Its presence also affected other proteins that seem to be involved in fibrosis.

**What Do These Findings Mean?**

Osteopontin may have a key role in the pathway that causes fibrosis to occur in the lungs of people with idiopathic pulmonary fibrosis. Further work will need to be done to confirm these results, but in the future drugs directed against osteopontin or one of the related proteins might be a possible treatment for the disease. Currently there are no such drugs. Additionally osteopontin may be useful in the diagnosis and early detection of the disease, but further studies are required.

**Where Can I Get More Information Online?**

Medline Plus has links to many pages with information on the disease:

http://www.nlm.nih.gov/medlineplus/pulmonaryfibrosis.html

The Coalition for Pulmonary Fibrosis is a nonprofit organization that has information for patients as well as physicians:

http://www.coalitionforpf.org/

The Dorothy P. and Richard P. Simmons Center for Interstitial Lung Diseases contains information about IPF for patients and their families:

http://simmonscenterild.upmc.com/

## Abbreviations

APMA: aminophenylmercuric acetate
BAL: bronchoalveolar lavage
EGFR: epidermal growth factor (receptor)
IPF: idiopathic pulmonary fibrosis
MMP: matrix metalloprotease
SD: standard deviation
TGF-β1: transforming growth factor-beta 1
TIMP: tissue inhibitor of metalloprotease
